# Supplementary figures and images for: Adhesion-regulated junction slippage controls cell intercalation dynamics in an Apposed-Cortex Adhesion Model
Source: PLoS Comput Biol. 2022 Jan 28;18(1):e1009812. doi: 10.1371/journal.pcbi.1009812 (PMC8887740; doi:10.1371/journal.pcbi.1009812)

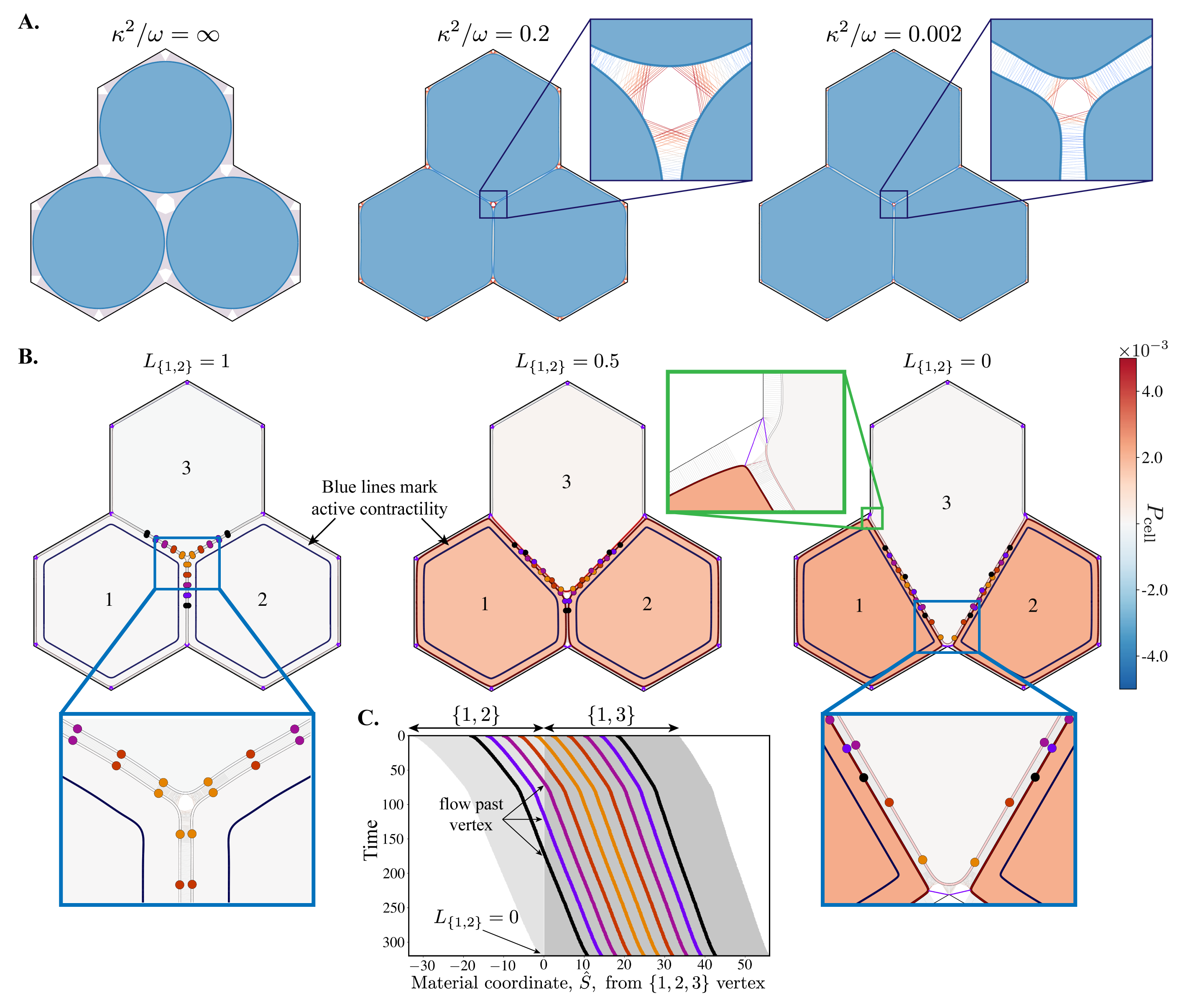

Supplement: S1 Fig — (A) Representative 3-cell tissues across parameter space. The right-most example is the κ2/ω = 0.002 isoline with coloured circles in Fig 2B. (B) Snapshots from Movie 2B, driving junction shrinkage with asymmetric contractility. The whole cortices of cells 1 and 2 are contractile (dark blue line in cells shows where active contractility has been applied). Coloured dots represent fixed material (Lagrangian) points that flow past one another, between apposed cortices, demonstrating cell–cell slippage. Boundary vertices have been pinned with extra-stiff adhesions (50ω; purple lines highlighted in green box) to maintain vertices at boundary angular points. Cell shading represents the magnitude of isotropic cell stress Pcell. (C) Kymograph along junctions {1, 2} and {1, 3} in the cortex of cell 1, showing the motion of material points (coloured Lagrangian markers) during the simulation shown in B and S2 Movie. The x-axis origin is at the transition from the {1, 2} to {1, 3} junction. Grey shading represents the extent of each junction, with darker grey showing growing {1, 3} and lighter showing the shrinking {1, 2}. Coloured lines crossing the boundary between light/dark grey shading indicate material points flowing past the vertex, from {1, 3} to {1, 2}. (TIF) [file pcbi.1009812.s014.tif]

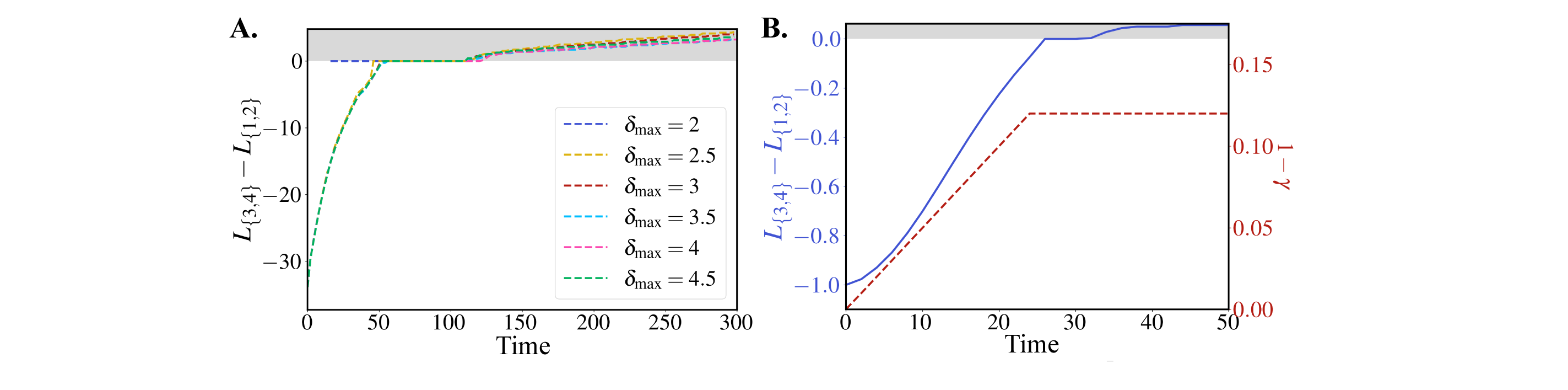

Supplement: S2 Fig — (A) Signed length of T1 junction vs. simulation time-step (as Fig 5A) for a range of δmax, with (κ, ω) = (0.01, 0.05), τadh = 10τcor, γ = 1 − 0.04 and δγ = 4. The dynamics are robust, up to δmax ≤ 2 where the {1, 2} junction fully fractures at simulation step 15 See S12 Movie. (B) Signed length of T1 junction vs. simulation time-step where the magnitude of active contractility (1 − γ; dashed red line) increases linearly over simulation time (see also S13 Movie). Taking the cortical timescale as τadh = 50s, we infer the total shrinkage time, T ∼ 20 mins. All other parameters match A, with δmax = 4. (TIF) [file pcbi.1009812.s015.tif]

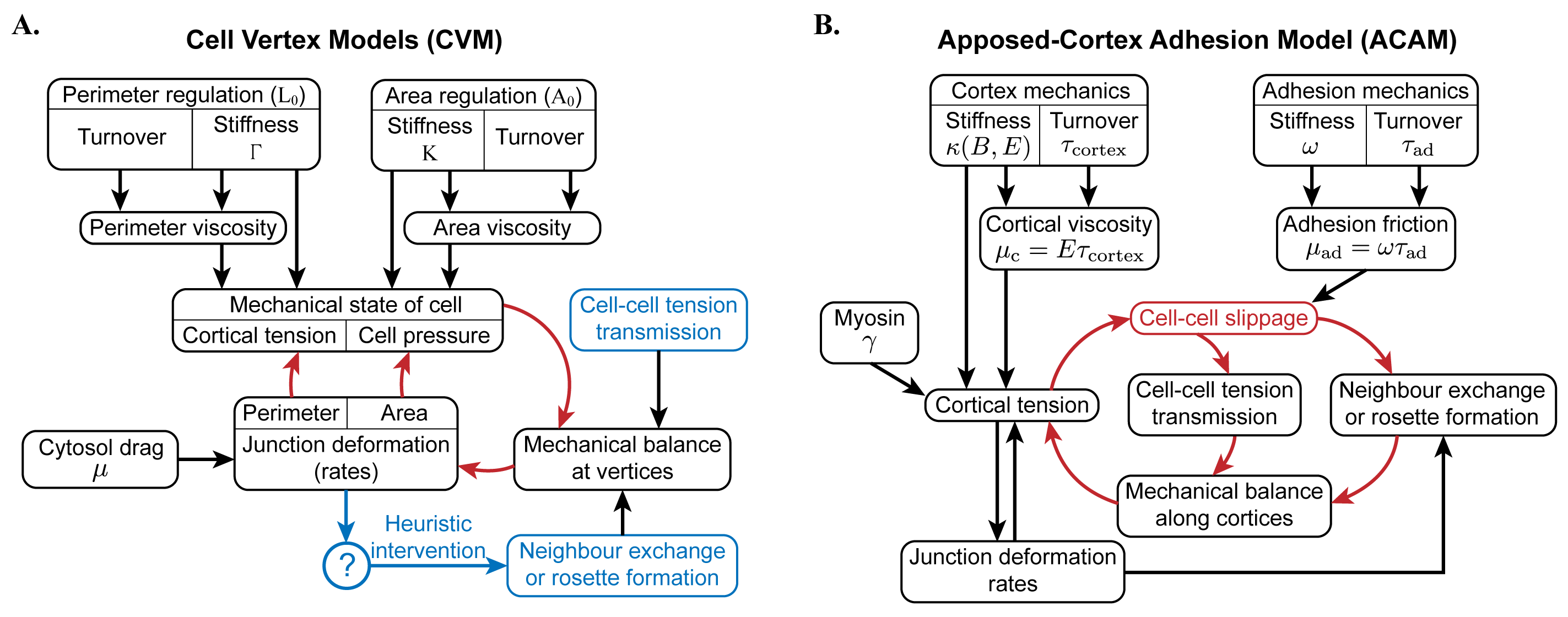

Supplement: S3 Fig — Notice that, for the ACAM, all parent nodes are linked to model parameters that regulate subcellualr properties, such that all derived cell-level behaviours are can be traced back to subcellular mechanics. For the CVM, cell-cell tension transmission and neighbour exchange behaviours cannot be regulated in the model. Furthermore, the parent nodes of the CVM relate to cell-level, rather than subcellular, properties. Red arrows highlight loops in the network. (TIF) [file pcbi.1009812.s016.tif]
