# Supplementary material for: Adhesion-regulated junction slippage controls cell intercalation dynamics in an Apposed-Cortex Adhesion Model
Source: PLoS Comput Biol. 2022 Jan 28;18(1):e1009812. doi: 10.1371/journal.pcbi.1009812 (PMC8887740; doi:10.1371/journal.pcbi.1009812)
Supplement: S1 Table — † For this simulation with asymmetric contractility across some bicellular junctions, cells 1 and 2 were given γ = 1 − 0.01 = 0.99 and cells 3 and 4 were set with γ = 1 − 0.0025 = 0.9975. (PDF) [file pcbi.1009812.s017.pdf]

**Simulation**	**Cortex stiffness** κ	**Adhesion stiffness** ω	**Adhesion turnover** τ_{adh}	**Active contractility** γ	**Background contractility** γ_0	**Pre-stress cell pairs**	**Max adhesion length** $\delta_{\max}$
Movie 1A	0.01	0.05	τ_{cor}	$\{1,\ldots,0.9\}$	1	$\{1,2\},\{1,3\},\{2,3\}$	4
Movie 1B	0.01	0.05	τ_{cor}	$1-0.005$	1	$\{1,2\}$	4
Movie 2A	0.01	0.05	$10\,\tau_{\mathrm{cor}}$	$1-0.04$	$1{-}2\times10^{-4}$	$\{1,2\}$	4
Movie 3A	0.01	0.05	$<\tau_{\mathrm{cor}}$	$1-0.04$	$1{-}2\times10^{-4}$	$\{1,2\}$	4
Movie 3B	0.01	0.05	$<\tau_{\mathrm{cor}}$	$1-0.04$	$1{-}2\times10^{-4}$	$\{1,2\}$	4
Movie 3C	0.01	0.05	$10\,\tau_{\mathrm{cor}}$	$1-0.04$	$1{-}2\times10^{-4}$	$\{1,2\}$	4
Movie 3D	0.01	0.05	$<\tau_{\mathrm{cor}}$	$1-0.04$	$1{-}2\times10^{-4}$	$\{1,2\}$	4
Movie 3E	0.01	0.05	$10\,\tau_{\mathrm{cor}}$	$1-0.04$	$1{-}2\times10^{-4}$	$\{1,2\}$	4
Movie 4A	0.01	0.05	$10\,\tau_{\mathrm{cor}}$	$1-0.01$ & $1-0.0025^{\dagger}$	$1{-}2\times10^{-4}$	$\{1,2\},\{1,3\},\{1,4\},\{2,3\},\{2,4\}$	4
Movie 5A	0.01	0.05	τ_{cor}	$1-0.04$	$1{-}2\times10^{-4}$	$\{1,2\},\{1,3\}$	4
Movie 5B	0.01	0.05	$100\,\tau_{\mathrm{cor}}$	$1-0.04$	$1{-}2\times10^{-4}$	$\{1,2\},\{1,3\}$	4
S1 Movie	0.01	0.05	$10\,\tau_{\mathrm{cor}}$	$1-0.04$	$1{-}2\times10^{-4}$	$\{1,2\}$	2
S2 Movie	0.01	0.05	$10\,\tau_{\mathrm{cor}}$	γ decreasing	$1{-}2\times10^{-4}$	$\{1,2\}$	4
