# Supplementary material for: Adhesion-regulated junction slippage controls cell intercalation dynamics in an Apposed-Cortex Adhesion Model
Source: PLoS Comput Biol. 2022 Jan 28;18(1):e1009812. doi: 10.1371/journal.pcbi.1009812 (PMC8887740; doi:10.1371/journal.pcbi.1009812)
Supplement: S1 Text — Document providing mathematical derivation of the ACAM. (PDF) [file pcbi.1009812.s018.pdf]

SUPPLEMENTARY DOCUMENT

PREPRINT

Alexander Nestor-Bergmann[1*], Guy B. Blanchard[1], Nathan Hervieux[1], Alexander G. Fletcher[3], Jocelyn Étienne[2*], and Bénédicte Sanson[1]

[1] *Department of Physiology, Development and Neuroscience , University of Cambridge , Cambridge, United Kingdom*
[2] *Univ. Grenoble Alpes, CNRS , LIPHY , F-38000 Grenoble, France*
[3] *School of Mathematics and Statistics and Bateson Centre , University of Sheffield , Sheffield, United Kingdom*

January 18, 2022

* Corresponding authors: an529@cam.ac.uk (ANB); jocelyn.etienne@univ-grenoble-alpes.fr (JE)

This document outlines a mechanical model of an epithelial tissue with active material properties. In a departure from many previous models, we represent the junctional cortices of individual cells explicitly, with adhesion forces acting between the cortices of neighbouring cells.

Geometry and kinematics

The cell cortex is modelled as a morphoelastic rod. We consider three configurations for the cortex (Figure 1D):

- the *initial* configuration, $\hat{\mathscr{C}}$, parameterised by a Lagrangian coordinate, $\hat{S} \in [0, \hat{L}]$, representing the arc length. This is the configuration that is adopted by the cortex when the cortex is undeformed, with neither external forces or internal active stress.
- The *reference* (virtual) configuration, $\mathscr{C}$, parameterised by $S \in [0, L]$. This is the unstressed configuration adopted by the cortex at time t. It is a conceptual configuration that the cortex adopts in response to active (pre-) stresses, but in the absence of external forces and boundary conditions. Due to physical constraints, the reference configuration may not be physically realisable in Euclidean space and can be defined only locally. For example, active stresses in the cortex may lead to a self-intersecting geometry, but this would be prevented in physical space.
- The *current* configuration, $\mathscr{c}$, parameterised by $s \in [0, l]$. This is the actual configuration that is adopted by the cortex in physical space, balancing pre-stresses, body loads and boundary conditions.

These configurations are related through a multiplicative decomposition. Material elements in the cortex are taken from the initial, undeformed configuration, $\hat{\mathscr{C}}$, to the reference (virtual) configuration, $\mathscr{C}$, by an active stretch:

$$\gamma = \frac{\partial S}{\partial \hat{S}}. \tag{1}$$

The active stretch represents local changes in material length, with $\gamma > 1$ for active growth and $\gamma < 1$ representing active contraction. Setting $\gamma < 1$ represents active contractility in the cortex, for example modelling the effect of bound myosin motors on the actin cytoskeleton. The mechanical effect of γ is to impose a residual stress, or pre-stress, in the cortex. Elastic stresses are generated when the cortex is subjected to external loading and boundary conditions, taking material elements from the virtual, $\mathscr{C}$, configuration to current, $\mathscr{c}$, configuration via an elastic stretch:

$$\alpha = \frac{\partial s}{\partial S}. \tag{2}$$

The total stretch, λ, between the initial and current configuration then satisfies

$$\lambda = \gamma\alpha \quad \iff \quad \frac{\partial s}{\partial \hat{S}} = \frac{\partial S}{\partial \hat{S}}\frac{\partial s}{\partial S}. \tag{3}$$

This decomposition is illustrated in Figure 1D.

In modelling the cortex as a morphoelastic rod, we follow the notation [1] and Chapter 6 in particular. Starting from the full 3D description, we provide simplified 2D equations describing the mechanics of the cortex in the apical plane. Under the rod representation, the cell cortex is defined geometrically by the position of the centre line, $\mathbf{r}(S,t)$, relative to the reference frame, mapping S to the fixed Cartesian basis $\{\mathbf{e}_1, \mathbf{e}_2, \mathbf{e}_3\}$ via $\mathbf{r} = x\mathbf{e}_1 + y\mathbf{e}_2 + z\mathbf{e}_3$. In the general frame, the rod is characterised by an orthonormal basis, $\{\mathbf{d}_1, \mathbf{d}_2, \mathbf{d}_3\}$. We set $\mathbf{d}_3$ to be aligned with the tangent of the centre line, normal to the rod cross section, and $\mathbf{d}_1$ and $\mathbf{d}_2$ are chosen to lie in the principal directions of the cross section, with $\mathbf{d}_3 = \mathbf{d}_1 \times \mathbf{d}_2$. The full kinematic description of the cortex is given by

$$\frac{\partial \mathbf{r}}{\partial S} = \mathbf{v}, \tag{4a}$$

$$\frac{\partial \mathbf{d}_i}{\partial S} = \mathbf{u} \times \mathbf{d}_i \quad \text{for } i = 1, 2, 3, \tag{4b}$$

where $\mathbf{v}$ is a stretch vector ($|\mathbf{v}| > 0$) and $\mathbf{u}$ is the Darboux vector. We detail these quantities under our specific assumptions below.

We simplify the generalised three-dimensional formulation with some key assumptions. Since epithelial dynamics are often driven in the apical plane, we model only the apical cortex and assume that it remains planar (torsion free). Furthermore, we assume that the cortex is unshearable, has a circular cross section and is naturally straight (no reference curvature). Importantly, however, the cortex is extensible. Under these conditions, $\mathbf{d}_3 = \cos\theta\mathbf{e}_1 + \sin\theta\mathbf{e}_2$ represents

the rod tangent, $\mathbf{d}_1$ is the rod normal and $\mathbf{d}_2 = \mathbf{e}_z$. The angle, $\theta(S)$, is the deflection of the cortex relative to the $\mathbf{e}_1$ axis. From these conditions, we have [1]

$$\mathbf{v} = \alpha \mathbf{d}_3, \tag{5a}$$
$$\mathbf{u} = (0, u_2, 0), \tag{5b}$$

where $u_2 = \alpha \frac{\partial \theta}{\partial s} = \frac{\partial \theta}{\partial S}$ is related to the Frenet curvature, $\frac{\partial \theta}{\partial s}$, of the cortex. Thus, the full kinematic description (4) is simplified to

$$\mathbf{r}' = \alpha \mathbf{d}_3, \tag{6a}$$
$$\mathbf{d}_1' = -u_2 \mathbf{d}_3, \tag{6b}$$
$$\mathbf{d}_2' = \mathbf{0}, \tag{6c}$$
$$\mathbf{d}_3' = u_2 \mathbf{d}_1, \tag{6d}$$

where derivatives w.r.t. S (virtual) are denoted $(\cdot)'$.

Cortex mechanics

With respect to the reference (virtual) configuration the balance of linear and angular momentum in the cortex give

$$\mathbf{n}' + \mathbf{n}_{\text{ext}} = \mathbf{0}, \tag{7a}$$
$$\mathbf{m}' + \mathbf{r}' \times \mathbf{n} + \mathbf{m}_{\text{ext}} = \mathbf{0} \tag{7b}$$

where $\mathbf{n}(S)$ is the force in a cross-section of the cortex, $\mathbf{n}_{\text{ext}}$ is the external force, $\mathbf{m}(S)$ is the moment and $\mathbf{m}_{\text{ext}}$ is the external moment. Following our assumptions above, the cortex behaves as a planar rod, extensible in the tangential $\mathbf{d}_3$ direction only, such that the force can be written

$$\mathbf{n} = n_1 \mathbf{d}_1 + E(\alpha - 1)\mathbf{d}_3, \tag{8}$$

where E is the extensional modulus of the cortex and n_1 is unknown. The cortex moment is reduced to

$$\mathbf{m} = B u_2 \mathbf{d}_2, \tag{9}$$

where B is the bending modulus of the cortex. Using (8) and (9) with (7b), assuming no external moment, we find the component of force normal to the cortex

$$\begin{aligned} n_1 &= -\frac{B}{\alpha} u_2' \\ &= -\frac{B}{\alpha} \theta''. \end{aligned} \tag{10}$$

Then, the balance of linear momentum (7a) gives

$$\begin{aligned} \mathbf{n}' &= \left(-\frac{B\theta''}{\alpha} \mathbf{d}_1 + E(\alpha - 1)\mathbf{d}_3 \right)' \\ &= \left(-\frac{B\theta'''}{\alpha} + \frac{B\alpha'\theta''}{\alpha^2} + E(\alpha - 1)\theta' \right) \mathbf{d}_1 + \left(\frac{B\theta'\theta''}{\alpha} + E\alpha' \right) \mathbf{d}_3 \\ &= -\mathbf{f}_{\text{ext}} \end{aligned} \tag{11}$$

Thus we have

$$-\frac{B\theta'''}{\alpha} + \frac{B\alpha'\theta''}{\alpha^2} + E(\alpha - 1)\theta' = -\mathbf{f}_{\text{ext}} \cdot \mathbf{d}_1, \tag{12a}$$
$$\frac{B\theta'\theta''}{\alpha} + E\alpha' = -\mathbf{f}_{\text{ext}} \cdot \mathbf{d}_3. \tag{12b}$$

These equations describe the position of the cortex in the deformed configuration relative to the reference (virtual) configuration. However, for our purposes, it is more convenient to work with reference to the initial, undeformed configuration, upon which we can impose the active pre-stresses. We can make the change of variables, writing

$$\theta' = \frac{1}{\gamma} \theta^* \tag{13}$$

and

$$\theta'' = \frac{1}{\gamma}\left(\frac{1}{\gamma}\theta^*\right)^* = \frac{\theta^{**}}{\gamma^2} - \frac{\gamma^*\theta^*}{\gamma^3} \tag{14}$$

where $(\cdot)^* = \partial(\cdot)/\partial S_0$. With respect to the undeformed configuration, we obtain a sixth-order system with four unknowns $(\theta,\, \alpha,\, x, y)$:
Find $(\theta,\, \alpha,\, x, y) : s \in [0, l] \to \mathbb{R}^4$ for which

$$\begin{aligned} B\gamma^2\alpha\theta^{***} &= B(3\gamma^*\gamma\alpha + \gamma^2\alpha^*)\theta^{**} + B(\gamma^{**}\gamma\alpha - 3(\gamma^*)^2\alpha - \alpha^*\gamma^*\gamma)\theta^* \\ &\quad + E\gamma^4\alpha^2(\alpha - 1)\theta^* + \gamma^5\alpha^2\,\mathbf{f}_{\text{ext}}(x, y)\cdot\mathbf{d}_1, &(15a)\\ E\gamma^3\alpha\alpha^* &= -B\gamma\theta^*\theta^{**} + B\gamma^*(\theta^*)^2 - \gamma^4\alpha\,\mathbf{f}_{\text{ext}}(x, y)\cdot\mathbf{d}_3, &(15b)\\ x^* &= \gamma\alpha\cos\theta, &(15c)\\ y^* &= \gamma\alpha\sin\theta, &(15d)\end{aligned}$$

where (15c) and (15d) provide the position of the cortex in the current configuration. The system (15) is closed with the following periodic end-point conditions: $(\theta^{**}, \theta^*, \theta, \alpha, x, y)|_{s=l} = (\theta^{**}, \theta^*, \theta, \alpha, x, y)|_{s=0} + (0, 0, 2\pi, 0, 0, 0)$.

Cell-level stress

We define the stress tensor, $\boldsymbol{\sigma}$, over the region, $\mathscr{A}$, of the cell enclosed by the cell boundary, $\mathscr{C}$. In the current configuration, the stress tensor is symmetric and divergence free in equilibrium, satisfying $\boldsymbol{\sigma} = \nabla\cdot(\mathbf{r}\otimes\boldsymbol{\sigma})$. Taking an integral over the cell area and applying the divergence theorem we have [2,3]

$$\int_{\mathscr{A}} \boldsymbol{\sigma} dA = \oint_{\mathscr{C}} \mathbf{r}\otimes\boldsymbol{\sigma}\cdot\mathbf{d}_1 \;\mathrm{d}S, \tag{16}$$

such that the cell-level stress tensor, averaged over the cell area, A, can be evaluated as

$$\boldsymbol{\sigma}_{\text{cell}} = \frac{1}{A}\int_0^L \mathbf{r}\otimes\mathbf{n}'\,\mathrm{d}S. \tag{17}$$

The symmetry of stress is assured by the moment balance. The trace of the stress tensor dictates the sign of its larger eigenvalue. We can therefore use the total cell pressure, $P_{\text{cell}} = -\mathrm{tr}(\boldsymbol{\sigma}_{\text{cell}})/2$, to characterise the state of stress within the cell: cells with $P_{\text{cell}} > 0$ ($P_{\text{cell}} < 0$) are under net tension (compression), with the principal axis of cell stress being tensile (compressive).

Timescales and cortical viscosity

The cortex relaxation timescale, $\tau_{\text{cor}} \sim 50\,\text{s}$, is largely driven by the timescale of actin turnover [4,5]. This timescale is much shorter than the timescale over which rearrangement occurs ($\sim 20\,\text{min}$ [6]). We therefore assume that the cortex behaves as a viscoelastic fluid and locally update the rest length of cortex segments to the current length, such that $\lambda = 1$, dissipating stretching energies and setting the simulation timestep equal to τ_{cor}. Dynamics in the system can then be driven by changes in the mechanical properties of cells (e.g. pre-strain, rest length and stiffness) or the boundary conditions.

Many discrete models, such as vertex-based models, impose friction directly on cell vertices or other discrete material points [7–9]. This is traditionally imposed as a dissipative drag with the substrate. However, there is often little justification that this is a dominant drag term in the system. Instead, we allow dissipation to occur through friction between neighbouring cortices due to adhesion connections, in a similar mechanism that is known to occur in cell-substrate interactions [10,11]. This dissipation is regulated by the timescale of adhesion molecules, τ_{adh} (discussed below).

External forces: cell-cell adhesions

We consider the nature of the distributed external body forces, $\mathbf{f}_{\text{ext}}$, acting on the cortex whose work must balance the variation of mechanical energy in the cortex at equilibrium.

Adhesions are a key mechanical feature that regulate tissue stability and are a primary source of external forces acting on cell cortices. We model adhesion complexes at an effective level, imposing forces that may be a composite of contributions from E-cadherin, α- and β-catenin, vinculin and other molecules linked to the adhesion complex. The adhesion complex, as a whole, is modelled as a Hookean spring, of rest length δ_0, with a maximum binding length $\delta_{\max}$.

Adhesion timescales

FRAP experiments have revealed that adhesion recovery times are fast ($\sim 20\,\mathrm{s}$ [12,13]) compared with the timescales of cortical relaxation and neighbour exchanges. We therefore impose that adhesion binding times in the model are fast, such that unbound cortex locations form new bonds instantaneously whenever there is a neighbouring cortex within $\delta_{\max}$. We allow for the possibility of connection to a node that already has another adhesion.

In addition to recovery time, there is an additional – less considered – timescale associated with adhesion molecules: the average lifetime of the adhesion complex, τ_{adh}. This timescale represents adhesion turnover, or the average time taken from binding to unbinding. It is notably distinct from, and more difficult to measure than, the adhesion recovery time. To our knowledge, there have been no explicit measurements of this additional timescale. However, the aggregated force from adhesions acting on the cortex will be strongly affected by it. By considering the magnitude of τ_{adh} relative to the timescale of the cortex, τ_{cor}, we will model two limiting cases of behaviour.

Distribution of adhesions

In the limit of a continuum where adhesions have a line density over each cortex, the configuration of adhesion can be represented by a density function $\rho(s, s')$ of two points S and S' belonging respectively to cortices $\mathscr{C}$ and $\mathscr{C}'$ to be linked with an adhesion. This density function varies in time due to binding and unbinding events. Between these events, as cortices deform, adhesion complexes remain attached to the same material point of the cortex: this is why ρ is written in terms of reference configuration coordinate S and not current configuration one s. Taking into account an unbinding rate $1/\tau_{\mathrm{adh}}$ and instantaneous rebinding, we reach Eq. (4) of main text:

$$\tau_{\mathrm{adh}} \frac{\partial \rho}{\partial t}(S_i, S_j; t) = \Phi_\beta(s_i(S_i), s_j(S_j)) - \rho(S_i, S_j; t) \tag{18}$$

where Φ_β is the probability of all possible connections, which takes into account the current configuration rather than the reference one. It is chosen as an exponential normalisation function, given by:

$$\Phi_\beta(s_i, s_j) = \begin{cases} \dfrac{\exp\left[-\beta \delta(s_i, s_j)\right]}{N_\beta} & \text{if } N_\beta \neq 0, \\ 0 & \text{else,} \end{cases} \tag{19}$$

where:

$$N_\beta = \sum_{\substack{1 \le k \le N_C \\ k \neq i}} \int_0^{l_j} H[\delta(s_i, s_k)] \exp\left[-\beta \delta(s_i, s_k)\right] \mathrm{d}s_k.$$

Here $H[\delta(s_i, s_j)]$ is a Heaviside step function, defined as

$$H[\delta] = \begin{cases} 1 & \text{if } \delta \le \delta_{\max} \\ 0 & \text{otherwise,} \end{cases} \tag{20}$$

which removes the possibility of adhesion connections at a distance greater than $\delta_{\max}$. This takes the exponential ratio of the length of a single adhesion over the sum of the lengths of all adhesions connected to s_i. The normalisation function thereby uses the length of each possible adhesion to generate a probability of connection, which is used as a scaling factor to decrease the contribution from connections to cortices that are far away. The parameter $\beta > 0$ controls the bias of the function, where larger values produce a stronger bias to adhesions that are nearby. In the limit $\beta \to \infty$ the nearest adhesion connection is the only possible one. For $\beta = 1$ we have the conventional softmax function in base e and for $\beta = 0$ we have a uniform distribution. However, we suggest a value of $\beta \geq 10$, as smaller values do not provide enough bias, leading to nearby adhesions having the weakest contribution to the total force.

Adhesive force

Numerically, we represent each adhesion as a single spring-like bond at a discretisation point, weighting its force by the bond density around that point. Sufficiently far from cell vertices, the cortices of neighbouring cells are straight and

parallel, and the spacing between them in the current configuration is δ_0. Let us consider two neighbouring cells, i and j. At every position of coordinate s_i on the cortex of cell i, an adhesion may connect it to an available binding location on cortex j, at coordinate s_j, within a distance $\delta_{\max}$. The line force density from a single connection between s_i and s_j is

$$\mathbf{f}_{\text{spring}}(s_i, s_j) = \omega\,(\delta(s_i, s_j) - \delta_0)\,\hat{\mathbf{r}}(s_i, s_j) H[\delta(s_i, s_j)], \tag{21}$$

where ω is the product of the stiffness of a single adhesion bond and the line density of bonds, $\delta(s_i, s_j) = |\mathbf{r}(s_j) - \mathbf{r}(s_i)|$ is the distance between cortex coordinates s_i and s_j and $\hat{\mathbf{r}}(s_i, s_j) = (\mathbf{r}(s_j) - \mathbf{r}(s_i))/\delta(s_i, s_j)$ is a unit vector pointing from s_i to s_j. We see that the adhesion bonds strongly couple the equilibrium equations of neighbouring cells (12).

Slow adhesions: $\tau_{\text{adh}} \geq \tau_{\text{cor}}$. In this case, cortical turnover is the fastest timescale and explicit adhesion bonds connect two neighbouring cortices, keeping those portions of cortical material coupled across simulation time. Every bond is tracked and can persist for multiple timesteps, with an average fraction $1 - \tau_{\text{cor}}/\tau_{\text{adh}}$ turning over every time step, which corresponds to a stochastic implementation of Eq. (18). An adhesion bond pairing position s_i on cortex i to s_j on cortex j exerts then a force

$$\mathbf{f}_{\text{adh}} = \mathbf{f}_{\text{spring}} \Delta s_i \Delta s_j, \tag{22}$$

where the force is averaged over the segment lengths, Δs_i and Δs_j. The total force acting at s_i on cortex i is then the sum of adhesion force to the set $\mathscr{A}(s_i)$ of all points s_j connected to that location :

$$\mathbf{f}_{\text{ext}} = \sum_{s_j \in \mathscr{A}(s_i)} \mathbf{f}_{\text{adh}}(s_i, s_j). \tag{23}$$

When a location s_i on the cortex does not have an adhesion connection (and only in this case), a new bond is formed to the nearest neighbouring cortex location s_i^* within $\delta_{\max}$, if such a point exists. This corresponds to $\beta \to \infty$ above, and results in incrementing $\mathscr{A}(s_i^*)$ by one (and is the only occasion of increasing $\mathscr{A}$).

Fast adhesions: $\tau_{\text{adh}} \ll \tau_{\text{cor}}$. When adhesion turnover is the fastest timescale, we have the limit where adhesion binding is always in dynamic equilibrium, thus Eq. (18) simplifies to $\rho(S_i, S_j; t) = \Phi_\beta(s_i(S_i), s_j(S_j))$. In this case, the adhesion force at a particular cortex location is the mean-field (average) force from all possible bindings to neighbouring cortices within $\delta_{\max}$. In this regime, individual adhesion bonds need not be tracked. Instead, the cumulative adhesion force acting on the cortex is calculated based on the position of neighbouring cortices. Considering a tissue comprising N_C cells, labelled $i = 1, \ldots, N_C$, we define the total adhesion force at cortex coordinate s_i, on cell i, as the deterministic mean-field from all possible connections to neighbouring cortices within $\delta_{\max}$:

$$\mathbf{f}_{\text{ext}}(s_i) = \sum_{\substack{1 \leq j \leq N_C \\ j \neq i}} \int_0^{l_j} \Phi_\beta(s_i, s_j) \mathbf{f}_{\text{spring}}(s_i, s_j)\, \mathrm{d}s_j. \tag{24}$$

The sum is over all other cells in the tissue, but the density function is zero for points located farther than $\delta_{\max}$. Figure 1C provides an example of the possible adhesions connected to a point on the cortex, at a vertex, and the scaled force produced by the adhesions under (24).

Nondimensional governing equations

We nondimensionalise lengths on the adhesion rest length (equivalent to the bicellular spacing), δ_0, and use

$$\delta = \delta_0 \tilde{\delta}, \qquad \omega = E\tilde{\omega}, \qquad \tau = E\tilde{\tau}, \tag{25}$$

using tildes to denote dimensionless variables. The dimensionless force balance in the normal and tangential directions (per unit S) is then

$$-\frac{\kappa\theta'''}{\alpha} + \frac{\kappa\alpha'\theta''}{\alpha^2} + (\alpha - 1)\theta' = -\mathbf{f}_{\text{ext}} \cdot \mathbf{d}_1, \tag{26a}$$

$$\alpha' + \frac{\kappa\theta'\theta''}{\alpha} = -\mathbf{f}_{\text{ext}} \cdot \mathbf{d}_3. \tag{26b}$$

where $\tilde{\kappa} = \sqrt{B/E}/\delta_0$ is a nondimensional parameter corresponding to the relative length scale over which bending affects the cortex around the vertex. The dimensionless force from a single adhesion bond is

$$\tilde{\mathbf{f}}_{\text{spring}}(\tilde{s}_i, \tilde{s}_j) = \tilde{\omega}\left(\tilde{\delta}(\tilde{s}_i, \tilde{s}_j) - 1\right) \hat{\mathbf{r}}(\tilde{s}_i, \tilde{s}_j) H[\tilde{\delta}(\tilde{s}_i, \tilde{s}_j)]. \tag{27}$$

Solving with respect to the deformed configuration, we can define a dimensionless state vector of unknowns $\mathbf{\Psi}(s) = (\theta^{**}, \theta^{*}, \theta, \alpha, \tilde{x}, \tilde{y})$. From (15), we are required to solve:

Find $\mathbf{\Psi} : s \in [0, \tilde{l}] \to \mathbb{R}^6$ such that

$$\mathbf{\Psi}^* = \begin{pmatrix} F_\theta(\psi_1, \psi_2, \psi_3, \psi_4, \psi_4^*, \psi_5, \psi_6) \\ \psi_1 \\ \psi_2 \\ F_\alpha(\psi_1, \psi_2, \psi_3, \psi_4, \psi_5, \psi_6) \\ \gamma\psi_4 \cos\psi_3 \\ \gamma\psi_4 \sin\psi_3 \end{pmatrix}, \tag{28}$$

with

$$\begin{aligned} F_\theta(\theta^{**}, \theta^*, \theta, \alpha, \alpha^*, \tilde{x}, \tilde{y}) \; &= \theta^{**}\left[3\gamma^*\gamma^{-1} + \alpha^*\alpha^{-1}\right] + \theta^*\left[\gamma^{**}\gamma^{-1} - 3(\gamma^*/\gamma)^2 - \alpha^*\gamma^*\gamma^{-1}\alpha^{-1}\right. \\ &\quad \left. + \kappa^{-1}\gamma^2\alpha(\alpha-1)\right] + \kappa^{-1}\gamma^3\alpha\, \mathbf{f}_{\text{ext}}(\tilde{x}, \tilde{y}) \cdot \mathbf{d}_1, \qquad \text{(29a)} \\ F_\alpha(\theta^{**}, \theta^*, \theta, \alpha, \tilde{x}, \tilde{y}) \; &= -\kappa\gamma^{-2}\alpha^{-1}\theta^*\theta^{**} + \kappa\gamma^*\gamma^{-3}\alpha^{-1}(\theta^*)^2 - \gamma\, \mathbf{f}_{\text{ext}}(\tilde{x}, \tilde{y}) \cdot \mathbf{d}_3, \qquad \text{(29b)} \end{aligned}$$

where $\mathbf{d}_3 = \cos\theta\mathbf{e}_1 + \sin\theta\mathbf{e}_2$ and $\mathbf{d}_1 = -\sin\theta\mathbf{e}_1 + \cos\theta\mathbf{e}_2$. The system is subject to periodic boundary conditions $\mathbf{\Psi}(\tilde{l}) = \mathbf{\Psi}(0) + (0, 0, 2\pi, 0, 0, 0)^T$.

Parameter selection

When initialising a cell, we must set a dimensionless reference length, relative to the spacing between apposed cortices (set to 1 in the nondimensionalisation). In the *Drosophila* germ-band, cells have a radius of approximately $3.5\,\mu\text{m}$ prior to gastrulation [6,14]. Conversely, cells in the developing wing disc epithelium have a smaller radius of $\sim 1 - 3\,\mu\text{m}$ (based on area measurements of $5 - 30\,\mu\text{m}^2$ [15]). Electron microscopy has found that the spacing between apposed cell cortices is approximately $30 - 40\,\text{nm}$ (estimated using scale bar in Figure 7 of [16]). These observations put the ratio of the cell radius to the bicellular spacing in the range $25 - 110$. Cells with larger radii are more computationally expensive to simulate. We therefore work in the lower bound and initialise cells (as a circle) with a dimensionless radius of 35, as a representative value.

It is difficult to measure the cortex extensional and bending moduli and the stiffness of adhesion bonds *in vivo*. Figure 2B of the main text demonstrates that the ratio of the dimensionless parameters, κ^2/ω, can be fitted to the size of openings around cell vertices. To our knowledge, due to difficulties in achieving the required resolution, these measurements have not yet been reported. Using STEAD microscopy, we do not observe significant openings around cell vertices. We therefore choose order-of-magnitude estimates $\kappa = 1 \times 10^{-2}$, $\omega = 5 \times 10^{-2}$, which give $\delta^{\text{vert}} \sim 1.43$, for this representative study.

Deriving the mechanical balance from the energetic formulation

In the main text, we present the model in terms of the mechanical energy (returning to the dimensional model):

$$\mathscr{U}(S) = \oint_C \left[\frac{1}{2}Bc(S)^2 + \frac{1}{2}E\varepsilon(S)^2\right] \mathrm{d}S, \tag{30}$$

where $c = \theta'$. We demonstrate that this is equivalent to the rod formulation described above by deriving the balance of linear and angular momentum in the cortex from $\mathscr{U}$. We follow a variational approach, considering an infinitesimal perturbation from an arbitrary configuration of the cortex. The first variation of the mechanical energy gives the forces and torques in the cortex and at the boundaries:

$$\delta\mathscr{U} = \left[\mathbf{m}\cdot\delta\mathbf{r}' + \mathbf{n}\cdot\delta\mathbf{r}\right]_0^L - \int_0^L \mathbf{n}'\cdot\delta\mathbf{r} + \mathbf{m}'\cdot\delta\mathbf{r}' \;\mathrm{d}S. \tag{31}$$

We derive these terms by taking the first variation of (30) to get

$$\delta\mathscr{U} = \int_0^L \left[Bc\delta c + E\varepsilon\delta\varepsilon\right] \mathrm{d}S. \tag{32}$$

Let us consider the bending and extensional terms separately. For the bending term, note that $c = \sqrt{\mathbf{d}_3' \cdot \mathbf{d}_3'}$ such that

$$\begin{aligned} \delta c &= \frac{\mathbf{d}_3' \cdot \delta \mathbf{d}_3'}{c} \\ &= \mathbf{d}_1 \cdot \delta \mathbf{d}_3'. \end{aligned} \tag{33}$$

Performing two rounds of integration by parts, we have

$$\begin{aligned} \int_0^L B c \delta c \ \mathrm{d}S &= \int_0^L B\theta' \mathbf{d}_1 \cdot \delta \mathbf{d}_3' \ \mathrm{d}S \\ &= [B\theta' \mathbf{d}_1 \cdot \delta \mathbf{d}_3]_0^L - \int_0^L B(\theta'' \mathbf{d}_1 - \theta'\theta' \mathbf{d}_3) \cdot \delta \mathbf{d}_3 \, \mathrm{d}S \\ &= [B\theta' \mathbf{d}_1 \cdot \delta \mathbf{d}_3]_0^L - \int_0^L \frac{B\theta''}{\alpha} \mathbf{d}_1 \cdot \delta \mathbf{r}' \, \mathrm{d}S \\ &= \left[B\theta' \mathbf{d}_1 \cdot \delta \mathbf{d}_3 - \frac{B\theta''}{\alpha} \mathbf{d}_1 \cdot \delta \mathbf{r} \right]_0^L + \int_0^L \left(\frac{B\theta''}{\alpha} \mathbf{d}_1 \right)' \cdot \delta \mathbf{r} \, \mathrm{d}S, \end{aligned} \tag{34}$$

where we have used the torsion-free kinematic identities (6) and the fact that $\alpha \delta \mathbf{d}_3 = \delta \mathbf{r}' - (\mathbf{d}_3 \cdot \delta \mathbf{r}')\mathbf{d}_3$. For the extensional term, we note that $\varepsilon = \sqrt{\mathbf{r}' \cdot \mathbf{r}'} - 1$ such that

$$\begin{aligned} \delta \varepsilon &= \frac{\mathbf{r}' \cdot \delta \mathbf{r}'}{\alpha} \\ &= \mathbf{d}_3 \cdot \delta \mathbf{r}'. \end{aligned} \tag{35}$$

Again, using integration by parts we have

$$\begin{aligned} \int_0^L E \varepsilon \delta \varepsilon \, \mathrm{d}S &= \int_0^L E(\alpha - 1) \mathbf{d}_3 \cdot \delta \mathbf{r}' \, \mathrm{d}S \\ &= [E(\alpha - 1) \cdot \delta \mathbf{r}]_0^L - \int_0^L (E(\alpha - 1)\mathbf{d}_3)' \cdot \delta \mathbf{r} \, \mathrm{d}S. \end{aligned} \tag{36}$$

Collecting terms in (34) and (36), referencing (31), we have no net moment and the force gradient in the cortex is given by

$$\mathbf{n}' = \left(-\frac{B\theta''}{\alpha} \mathbf{d}_1 + E(\alpha - 1)\mathbf{d}_3 \right)', \tag{37}$$

which is equivalent to (11). The terms evaluated at the boundaries impose moment and force continuity at the end-points.

Numerical implementation and pseudocode

A pseudocode implementation of the model is provided in Algorithm 1.

Algorithm 1: Apposed-cortex model pseudocode

```
Result: Simulate dynamics of tissue with given pre-strain rule
Read tissue from file, or initialise new tissue;
for step in number of timesteps do
    if τ_adhesion ≥ τ_cor then
        Prune adhesions with probability p = τ_cor/τ_adh;
        Add new adhesions to unpaired cortex nodes;
    end
    Viscous cortex length update: dŜ ⟵ ds (giving λ = 1);
    Determine pre-stretch γ;
    Apply pre-stretch: dS ⟵ γdŜ;
    while tissue not in equilibrium do
        if τ_adh ≪ τ_cor then
            Prune all adhesions;
            Add new adhesions to all cortex nodes ;
        end
        for cell in tissue do
            Solve (28) for cell;
        end
        Check tissue equilibrium;
    end
end
```

The initialisation of a new tissue can be done as described in the main text, by duplicating a single cell fitted within a hexagon. Alternatively, it is also possible to initialise multiple circles at randomised locations within a global stencil and relax them all simultaneously. However, this requires smaller changes in the adhesion strength since neighbouring cells move simultaneously and must be prevented from overlapping, thus it requires increased computational time and can be less numerically stable.

Simulations were performed in Python 3. The system (28) is solved using the `solve_bvp` function from the `Scipy` library [17]. The function performs discretisation using a fourth-order collocation algorithm. The collocation system is solved using a damped Newton method with an affine-invariant criterion function, as described in [18]. The equations for each cortex are solved in parallel, taking the current position of neighbouring cortices as possible binding locations. This process is repeated, updating the rest length of the cortices at every time step, until a global equilibrium is reached. Adaptive mesh refinement was used at every relaxation step, to ensure that node spacing remained numerically stable and fast. Additional nodes were added in regions where the mesh spacing was greater than $\tilde{\delta}_0/4$ and nodes were removed where the spacing was less than $\tilde{\delta}_0/20$. The model parameters used for the simulations are given in Table 1.

Source code for running the published simulations can be found at `github.com/Alexander-Nestor-Bergmann/appcom`. Documentation and quick-start tutorials can be be found at `appcom.readthedocs.io`.

References

1. Goriely, A. *The mathematics and mechanics of biological growth*. Vol. 45. Springer, 2017.
2. Batchelor, G. "The stress system in a suspension of force-free particles". In: *J. Fluid Mech.* 41.3 (1970), pp. 545–570.
3. Nestor-Bergmann, A., Goddard, G., Woolner, S., and Jensen, O. "Relating cell shape and mechanical stress in a spatially disordered epithelium using a vertex-based model". In: *Math. Med. Biol.* 35.Supplement_1 (2018), pp. i1–i27.
4. Tanaka, F. and Edwards, S. "Viscoelastic properties of physically crosslinked networks. 1. Transient network theory". In: *Macromolecules* 25.5 (1992), pp. 1516–1523.
5. Clément, R., Dehapiot, B., Collinet, C., Lecuit, T., and Lenne, P.-F. "Viscoelastic dissipation stabilizes cell shape changes during tissue morphogenesis". In: *Curr. Biol.* 27.20 (2017), pp. 3132–3142.
6. Finegan, T., Hervieux, N., Nestor-Bergmann, A., Fletcher, A., Blanchard, G., and Sanson, B. "The tricellular vertex-specific adhesion molecule Sidekick facilitates polarised cell intercalation during Drosophila axis extension". In: *PLoS Biol.* 17.12 (2019).
7. Fletcher, A., Osterfield, M., Baker, R., and Shvartsman, S. "Vertex models of epithelial morphogenesis". In: *Biophys. J.* 106.11 (2014), pp. 2291–2304.
8. Alt, S., Ganguly, P., and Salbreux, G. "Vertex models: from cell mechanics to tissue morphogenesis". In: *Philos. Trans. R. Soc. B* 372.1720 (2017), p. 20150520.
9. Gracia, M., Theis, S., Proag, A., Gay, G., Benassayag, C., and Suzanne, M. "Mechanical impact of epithelial-mesenchymal transition on epithelial morphogenesis in Drosophila". In: *Nat. Commun.* 10.1 (2019), pp. 1–17.
10. Tawada, K. and Sekimoto, K. "Protein friction exerted by motor enzymes through a weak-binding interaction". In: *Journal of theoretical biology* 150.2 (1991), pp. 193–200.
11. Reboux, S., Richardson, G., and Jensen, O. "Bond tilting and sliding friction in a model of cell adhesion". In: *Proceedings of the Royal Society A: Mathematical, Physical and Engineering Sciences* 464.2090 (2008), pp. 447–467.
12. Cavey, M., Rauzi, M., Lenne, P.-F., and Lecuit, T. "A two-tiered mechanism for stabilization and immobilization of E-cadherin". In: *Nature* 453.7196 (2008), pp. 751–756.
13. Iyer, K., Piscitello-Gómez, R., Paijmans, J., Jülicher, F., and Eaton, S. "Epithelial viscoelasticity is regulated by mechanosensitive E-cadherin turnover". In: *Curr. Biol.* 29.4 (2019), pp. 578–591.
14. Butler, L., Blanchard, G., Kabla, A., Lawrence, N., Welchman, D., Mahadevan, L., et al. "Cell shape changes indicate a role for extrinsic tensile forces in Drosophila germ-band extension". In: *Nat. Cell Biol.* 11.7 (2009), pp. 859–864.
15. Dye, N., Popović, M., Spannl, S., Etournay, R., Kainmüller, D., Ghosh, S., et al. "Cell dynamics underlying oriented growth of the Drosophila wing imaginal disc". In: *Development* 144.23 (2017), pp. 4406–4421.
16. Fristrom, D. "Septate junctions in imaginal disks of Drosophila: a model for the redistribution of septa during cell rearrangement". In: *J. Cell Biol.* 94.1 (1982), pp. 77–87.
17. Virtanen, P., Gommers, R., Oliphant, T. E., Haberland, M., Reddy, T., Cournapeau, D., et al. *SciPy 1.0: Fundamental Algorithms for Scientific Computing in Python*. 2020. DOI: 10.1038/s41592-019-0686-2.
18. Ascher, U., Mattheij, R., and Russell, R. *Numerical solution of boundary value problems for ordinary differential equations*. SIAM, 1995.
