# Supplementary material for: Adhesion-regulated junction slippage controls cell intercalation dynamics in an Apposed-Cortex Adhesion Model
Source: PLoS Comput Biol. 2022 Jan 28;18(1):e1009812. doi: 10.1371/journal.pcbi.1009812 (PMC8887740; doi:10.1371/journal.pcbi.1009812)
Supplement: Attachment — Submitted filename: response_to_reviewers.docx [file pcbi.1009812.s019.docx]

Response to reviews

First, we would like to thank all Reviewers and the Editor for their remarks and suggestions, which have been very helpful. They have led us to make a number of clarifications which, we believe, improve the manuscript. Note that whilst doing so, we have changed some mathematical notations, in particular for the reference configuration of a cell.

Editor's comments

In addition to the reviewers' reports, I would like to add these points that I would like the authors to address:
1) I could not see in eq.2 that the adhesion lowers the overall energy of the cell membrane.

The jump to our discretised implementation of adhesion did appear to be problematic. We have therefore worked to derive an expression for the total energy of the system, given now in equation 5, including the continuous form of adhesion energy of which our algorithm gives a discrete approximation. We believe this will improve the clarity of what our model encompasses, and we would like to thank the Editor for this suggestion.

2) Why don't they consider a force-dependent dissociation rate of the adhesion molecules, ie that they dissociate when stretched ? This is also the basis of the effective friction that such binding-stretching-unbinding dynamics induce between cells (and at cell-substrate).
See for example in the context of a particle-based monolayer model:
Garcia, Simon, et al. "Physics of active jamming during collective cellular motion in a monolayer." Proceedings of the National Academy of Sciences 112.50 (2015): 15314-15319.‏

The adhesion molecules we are modelling here are those present at adherens junctions in epithelia, which is principally composed of the E-Cadherin complex. How E-Cadherins respond to forces in vivo is not fully elucidated (see Lenne et al., Dev Cell (2021) <https://doi.org/10.1016/j.devcel.2020.12.018>) for a recent discussion), so there is not a straightforward relationship that we could include in our model.

Our model currently describes de-adhesion through the maximum range, δ_max, (beyond which adhesion energy is constant) and the probability density function based on the softmax function. This is a simple implementation of such behaviour because it corresponds to a threshold maximum force and to having less probability for longer bonds. We can consider this as a crude approximation of Bell’s model, without the positive feedback property. This is a convenient choice to easily disentangle the origin of the behaviours exhibited by the model. We thus kept this choice, with a minimal number of parameters, to keep the model general for this paper.

In future applications of the model, specific stress responses of adhesions could be implemented, but the properties will depend on the given tissue context. For example, in the tissue that interests us, the Drosophila germ-band, recent work suggests that E-cadherin bonds can exhibit catch-bond-like behaviour in response to normal forces, but slip-bond-like behaviour in response to shear forces (Kale et al., Nat Comm (2018) doi: [10.1038/s41467-018-07448-8](http://dx.doi.org/10.1038/s41467-018-07448-8)). In our model, we would expect the response to normal forces to increase the fracture-resistance of the tissue in response to active stresses, while the shear stress response may facilitate cell-cell slippage to help shear movements during cell rearrangements. In summary, the stress response is not entirely known, and likely to be tissue specific, but our model would be a useful framework to explore such behaviours.

In response to this comment, we have added additional discussion and references on our implementation of adhesion, particularly from line 144.

3) This work seems relevant to discuss, in the context of how this model could be applied to biological problems:
Dai, Wei, et al. "Tissue topography steers migrating Drosophila border cells." Science 370.6519 (2020): 987-990.

This interesting paper finds that border cells tend to migrate through tricellular rather than bicellular gaps in the nurse cell mass. This might be relevant to the behaviour of vertices in our model, which are more susceptible to fracture than bicellular junctions. We have added this reference in this context in the discussion (line 377).

Reviewer's Responses to Questions

Reviewer #1:

Summary

In this manuscript, Nestor-Bermann et al develop and present the Apposed-Cortex Adhesion Model (ACAM), a new cell-based biophysical model for tissues along with several results related to cell-cell adhesion and rearrangements, of particular importance in ensuring tissues are able to undergo remodelling during many developmental processes. Many existing biophysical models for tissues, in particular vertex models, model the interface between two cells as a single edge with adhesions connecting the two cells together not explicitly modelled. This means that cell rearrangements in these models do not consider the dynamic uncoupling between two interfaces, and instead a rearrangement is often triggered by hand through a geometric argument, such as when an edge becomes small.

In the Apposed-Cortex Adhesion Model, the apical surface of each individual cell is modelled as a viscoelastic loop with both a bending and stretch stiffness, that can actively contract. When two cell interfaces are near, adhesion bonds bind the two interfaces together. These bonds are modelled by springs, attempting to keep interfaces connected, but also undergo turnover, enabling larger scale remodelling of junctions and even uncoupling between two connected interfaces as in rearrangements. Additionally, the junction is described as a continuum, enabling more complex cell shapes than in vertex models and variation along individual junctions. Model parameters are tuned to experimental data, for example, adhesion binding and unbinding rates, and actomyosin cortex turnover. Additional experiments are performed to measure spacing between two apposed cell cortices to estimate adhesive bond lengths.

Using this model, they first study the steady state geometry of a simple 3-cell tissue, predicting the spacing between cells at vertices, or 3-way junctions, as a function of adhesive stiffness and contractility. When contractility is too high, the vertex fractures and cells lose adhesion between much of their interfaces. Next, they model the process of actively driven rearrangements by increasing tension between two apposed interfaces. The tension shrinks the apposed cell interfaces which then lose adhesion and then extend passively. During this process, the cells lose some area depending on the rate of adhesion turnover, which is also observed experimentally. Additionally, rosettes, or 4+ cell interfaces may be formed when an additional rearrangement is triggered before the first is fully resolved and the adhesion turnover time is high.

Overall, this paper develops a novel biophysical model for cells and tissues that includes a more detailed description of cell-cell interfaces by including separated cortices with adhesion molecules connecting them. I feel that this model, and expansion upon it, could help our understanding of the mechanical behaviour of cell-cell junctions and rearrangements, in particular when we are interested in finer details such as the mechanics of adhesions or heterogeneity along junctions. Subject to addressing the points below, I think this paper would be worthy of publication.

# We thank the referee for the thorough and engaged review of our work. We are appreciative of the detailed comments on the emergent physics of the system, as well as specifics of our implementation. Major Points 1. The results for vertex spacing and neighbour exchange are all reliant on breaking of adhesions between two cells, which depends on the parameter delta_max. Most other parameters are tuned to experiments but this seems to have been chosen arbitrarily. How do the key results of this paper depend on this parameter?

The maximum adhesion binding length, delta_max, is not readily available from existing experimental data, and likely depends on context. In our model, fracture occurs when adhesions are stretched beyond this length. However, the selected value does not strongly affect the key results of the paper, compared to e.g. the adhesion stiffness and strength of active contractilty, which determine separation between cells. To demonstrate this lack of sensitivity we provide an additional Figure S2, highlighting that the dynamics of junction shrinkage, resolution and extension are largely unaffected by variations in delta_max, except the case where d_max <= 2 and the tissue fractures. Note, however, that this fracture could be prevented by increasing the adhesion strength or decreasing the active contractility. Furthermore, interestingly, the tissue repairs the fracture as the adhesions re-zip the cells, but becomes jammed at a 4-way vertex as the adhesions cannot bridge the gap (see new Movie S1).

2. What defines the rest configuration of a cell? With no contraction, bending energy wants to straighten edges so expands the lengths, and this expansion is relaxed. Should cells then get infinitely bigger to reduce bending energy while relaxing stretching energy?

This is not the case with our modelling choice, which is that bending energy is written in the reference configuration, following e.g. Audoly & Pomeau, “Elasticity and Geometry: From Hair Curls to the Non-linear Response of Shells” (2010). If we had made the other choice of writing it in the current configuration, then a circular loop would indeed have had an elastic equilibrium with a slight stretch (of the order of the slenderness of the rod, implying that the difference between these modelling choices has to be negligible) which, coupled with elastic relaxation, could have led to the behaviour the Reviewer describes.

Note that, in applications of our model, bending energy remains of small magnitude and bending is localised in corner regions. This actually corresponds to the class of “elastic strings” described by Marigo & Meunier, *J. Elas.* (2006) doi: 10.1007/s10659-005-9036-y

3. Similarly, what determines the static size of cells? If the only energy is from bending at the corners, can cells scale their lengths without changing the shape of corners and also remain stable?

Another interesting thought experiment. We presume that the Reviewer takes an example in which corners are defined by an external shape to which the cortex adheres to. If that shape is deformed with its angles conserved, indeed the bending energy will return to its previous value while the elastic stretch will relax. Note that with our simple approach of viscoelasticity, we are limited to changes of external conditions which vary slower than the cortex relaxes.

4. The time scales between simulations in Fig 5B and 5C seem off. Each simulation timestep is 50s, so the total T1 would take about 300 minutes, compared to 30 minutes in experiments. Why are these differences so large?

The reviewer is correct to assume that we have τ_cortex ≥ 50s for our viscous cells. In this paper, we have not directly fitted the magnitude of active prestress to match the rates of deformation in the germ-band. We do this for convenience, since simulations are more stable and equilibrium easier to reach with smaller forces. However, we have now included an additional Figure S2B, and Movie S2, to demonstrate that the timescale of shrinkage can be matched to specific systems (here, the germ-band, with around 20 mins) by also tuning the active contractility. This strengthens our confidence that the model captures essential phenomena at play in epithelial tissue, and we thank the reviewer for raising this question.

Minor Points

1. There have been other models of cells as individual entities connected by springlike adhesions that would be worth comparison eg Nematbakhsh et al (2017)

We thank the reviewer for highlighting this relevant paper, which we now cite in the introduction.
2. I find the details of the fluid model of the cortex a bit unclear. At the start of each simulation step, the rest lengths are relaxed such that the stretch alpha = 0?

We apologise for the confusion. At each step, the total stretch is dissipated, such that lambda = 1 (ratio of deformed to undeformed lengths = 1), by updating lengths in the undeformed configuration (S_0) to the current configuration (s) via: ds -> dS_0. We have added this detail to the pseudocode algorithm and discussion of cortical turnover in the supplementary document.

3. How do adhesions decide which node to connect to when rebinding? Is it to all nearby nodes or only the closest?

In the case when τ_ad < τ_cortex, a probability density function is used to determine the mean field effect of binding and unbinding with all possible target sites. This probability density function is biased towards closer sites, with a parameter β (the robustness of results with respect to this parameter has been tested but is not shown). In the alternative however, we have chosen for simplicity that new bonds form with the closest available neighbour. We have added further details on our implementation of adhesions to the main text and Supplementary Document, including details of our discrete implementation (see specifically line 116 of the Supplementary Document).
4. I think the explanation for W saturating in Eq.2 (so that these bonds are effectively broken during force balance steps) should be mentioned in the main text for clarity.

We have clarified this effect when the energy is introduced in the main text.

5. In Movie 2A: the cells 1 and 2 remain contractile even after they lose adhesion to each other, which is needed to complete the T1. I thought they are contractile in places where they share adhesions, so why does this happen?

The contractility is in place whenever the cortices are within range of one another (distance smaller than δ_max) regardless of whether there remain adhesions between them. In Movie 2A, adhesions between cells 1 and 2 are lost through turnover (compare τ_ad=100τ_cortex in Fig 3Biii) and replaced with adhesions to closer cortices of cells 3 and 4 before the cells move apart by more than δ_max, which is why contractility remains applied. This contractility is necessary to push through the 4-way (see “stalling” in Fig 3B). Note that our implementation is not the only way to achieve persistent contractility. For example, we could consider that Myosin II has a de-loading timescale and so remains on the cell cortices as they move apart.

We have added reworded our discussion of this implementation in section 2.5 to make this more clear.

6. In Movie 3A: the T1 occurs very fast, from one frame to the next cells 1 and 2 completely lose adhesion and are moved large distances. Is this what is actually simulated? This seems like a big jump, though it may be due to completely relaxing forces each time step.

Indeed, that is the full jump between equilibria. Our model being based on Maxwell viscoelasticity, at short times cortices are purely elastic. If in addition, we are in the case of “fast adhesions” that are also assumed to rearrange instantaneously, elastic jumps can be observed. These dynamics could be regularised by introducing another dissipative term, such as a friction with a substrate. Estimates for this friction have been given both at cellular (Saha et al, Biophys J (2016)) and tissular scales (Dicko et al, PLOS Com Bio (2017)), with hydrodynamic length ranging from 15 to 100 µm for *C elegans*, zebrafish and *Drosophila* embryonic epithelia, which argues for a small but not altogether negligible effect. However, this is likely to be impactful only in the fast adhesion regime and, since we wanted to highlight the role of adhesion turnover as an unexplored player that explains some phenomena in epithelial dynamics, we have chosen not to include other friction terms.

7. In the T1 simulations, it appears that the high fast a_d limit gives you vertex model like behaviour, especially the “isogonal” transformations seen in Noll et al (2017), while slow a_d limit gives JVM like behaviour since there is no material flow and each edge acts elastically. This would be worth discussing since the ACAM can tune between these two models.
We agree that the fast adhesion limit resembles a traditional vertex model, with vertices being free to slide if the perimeter is conserved, and increasing tau_ad allows us to tune towards the limit of a JVM (which would be tau_ad >> the timestep). However, we are not yet in a position to make a direct comparison to the results of Noll et al. That paper discusses shared properties of static equilibria, surrounding the angles at vertices and the relationship of the ‘tension network’ to dual centre networks. Though they do this in a JVM (see text above their eqn 3), similar results have been derived for general force networks, including CVMs (see Jensen et al., Proc Roc A (2020) [doi: 10.1098/rspa.2019.0716](https://doi.org/10.1098/rspa.2019.0716)). We are not yet sure to what extent these properties of discrete networks hold in our model.

8. Why do the low tau_d limit material points on different apposed cells slide at different rates when their tensions are equal eg in Fig 4F?

Our understanding is that local tension does not give velocity, but is related to the velocity gradient in the viscous limit, corresponding to the dynamics seen in Movie 3B. Taking the example of cells 1 and 3, and their common junction, we can understand the mechanical balance of each: from the simulation, we see that while the tension equilibrates due to fast adhesions (Fig 4F, left), the material flows differ (movie 3B). This can be accounted for by the fact that, due to symmetry, the cortex of cell 3 cannot have in- or outflow at the vertex {1,2,3}. Conversely, at the same vertex, the cortex of cell 1 is flowing past the vertex because of the strong contractility in the junction between cells 1 and 2. These different conditions at the vertex explain that velocity will differ within junctions even if tension is the same.

9. Can slippage of points between cells be observed in the experimental T1 movies eg by tracking punctae of Myosin II?

Our existing experimental data, visualising Myosin II, are taken with x40 magnification, which does not give sufficient resolution to accurately track punctae. In future, we could indeed aim to do this tracking in movies with higher spatial (and also temporal) resolution.

10. Why are the patterns of tension required to resolve T1s quickly as in Figure 5C? Are they resolved quickly when cell tension is increased as in vertex models?

The patterns of tension we show are not required to match the dynamics of shrinkage (see the new simulation discussed in Major point 4), with stronger tension in a single junction. However, we propose this cable-like structure as a reference to the situation that may arise in the germ-band, to help to speed up the extension phase. Of course, tissue-level forces and forces from medial Myosin (Collinet et al, NCB, (2015)) may also be contributing factors to be incorporated in more tissue-specific adaptations of this model.

As a general point, patterned contractility does not appear to be required to resolve T1s e.g. in the notum and wing disc tissues where there is no obvious patterned contractility; see  Curran et al., Dev Cell (2017) doi: 10.1016/j.devcel.2017.09.018. where they claim *“We find that neighbor exchange events are driven by stochastic fluctuations in junction length, regulated in part by junctional actomyosin.”*

We have added additional references that discuss different types of T1s across tissues.

11. Can 4 way vertices be stabilised with the use of tension?

As with rosettes, it appears that 4-way vertices can be stabilised. We have added this information to the manuscript. Please see question 12 for further discussion.

12. Why does tension stabilise rosettes in the high adhesion friction limit? Can the authors give an intuitive answer?

In the model, a stable rosette corresponds to an opened vertex such that new adhesions will not form between cortices which are not direct neighbours (e.g. cells 3 and 4 in Fig 3A, cells 7 and 2 in Fig 6). The opening of a vertex is determined by the bending rigidity and the cortical tension (Fig 2B and E), and also increases with the number of cells forming the vertex. Bending rigidity and cortical tension determine the maximal local curvature that any cortex will adopt and the number of cells involved in the vertex add an increase in the opening, scaling linearly with the number of cells, by a geometric effect. Thus, small values of tension can stabilise the 4-way junctions and rosettes by increasing the opening (preventing new adhesions across them) and, with equal tension, a symmetrical rosette will be more stable than a 4-way junction.

Note that this corresponds to metastability rather than to stability in the sense of Spencer et al, Eur Phys J E (2017) (<https://doi.org/10.1140/epje/i2017-11489-4>).

We have given some explanation of this in sections 2.6 and 2.11 and thank the reviewer for suggesting such an intuitive understanding of the higher order vertex stability.

13. Supplementary material line 52: “update the current length of the cortex segments to the current length”. Should this be “rest length to the current length”?

It should. Thank you.

## Reviewer #2:

The authors propose a novel mechanistic model for epithelial dynamics – specifically cell intercalation – where the key feature of the new model is the representation of cell-cell junctions not as single edges, but explicitly considering the apposed cell cortices of the two participating cells facing each other at that edge, where the individual junctional actomyosin cortex of each cell is modeled as a continuum viscoelastic rope-loop.

Let me start with the positives: I think this model is highly original and potentially valuable to the field. There are only very few published papers that consider any distinct contributions of the apposed cortices of the two participating cells, so that this Apposed-Cortex-Adhesion Model has the potential to make significant contributions to our understanding of intercalation; I think that there are a number of processes in intercalation that could involve some type of ‘heterotypic’ interaction between cortices. In terms of the theoretical modeling, the basic model is well crafted and well documented. Also, I wanted to mention that I thought the supplementary movies of the simulations were great, and I really enjoyed having data presented in this way.

We thank the reviewer for these positive comments. These are exactly the arguments that motivated us to start the project.

However, there are a number of issues that dampened my enthusiasm. The central sticking point was that in its current form, the manuscript does not yet seem to make a very compelling case that this new (and more fine-grained) model actually provides novel insights into cellular mechanisms, specifically into the mechanisms of intercalation in the drosophila germ band. In some cases, the model merely reproduced existing knowledge (and doesn’t discuss in much detail how their findings fit in with the published literature); in other cases, the model oversimplified the biological system to the point of potentially rendering the simulation results meaningless to the biological mechanism of interest; so I would like to focus on those aspects of the model.

We are sorry that our intentions may not have been completely clear. We have added an introductory paragraph at the beginning of the results section to clarify our intentions before the model is introduced.

We recognize that we do not fully explore all behaviours associated with intercalation in the germ-band, such as medial Myosin and tissue-level boundary conditions. Our intention with this paper is to identify the minimal ingredients required for cell intercalation, revealing general principles, through a new model that:

- Is parametrised by the physical properties of the known subcellular players involved.
- Extends limitations of existing models of cell intercalation to explore the behaviours associated with apposed cortices.

While it is tempting to delve straight into the specifics of a particular tissue, we feel that such an advancement must be introduced carefully and in general/minimal form before specific questions about particular issues can be addressed, which is why the reader interested in the implications for the understanding of specific morphogenetic movements can rightly feel that we do not go in sufficient details for their biological system of interest. However, we do believe that the model brings interesting insights to intercalation, e.g. that a purely junctional model of actomyosin and adhesions can recapitulate intercalation, that apical cell area during intercalation can be modulated by low slippage between apposed cortices, and that rosette formation can be recapitulated under given conditions. Additionally, using a tissue-level model to show how the mechanical properties of adhesion molecules can specify a slippage friction that regulates deformation rates represents a broader contribution to our biophysical understanding of active tissues.

One of these simplifications occurs very early the manuscript and propagates from there: The authors state that “we therefore model a vertex as the geometric point where three or more cells are coupled by adhesions, rather than a material point”. But why? There is a solid body of evidence that tricellular junctions (both in mammalian systems and in drosophila) are specialized and molecularly distinct structures that do in fact mark the material point where three or more cells meet, and which likely organize true three-way adhesions (as opposed to a combination of bicellular adhesions); the manuscript’s authors obviously know this since they themselves have published on the role of sidekick in tricellular junctions. In this reviewer's opinion, the existence of tricellular connections would completely change the rationale (or even invalidate the entire premise) of Section 2.2, i.e. the energetic ‘competition’ between maximizing adhesion contact in sharp corners vs elastic penalty due to bending. Are there compelling arguments for ignoring the contributions of tricellular connections? Are the authors making the point that tricellular junction components act like combinations of bicellular junctions for the purposes of the model? This question deserves more than a passing mention in the Discussion section, and should be discussed in much greater detail when the rationale for the model components is explained.

These are very interesting points and certainly on our radar.

There is increasing evidence that vertices are mechanically distinct regions in a tissue, localising particular molecules such as Sdk or Canoe, which may have direct mechanical influence or modulate the properties of adhesions such as E-cadherin at vertices. Yet, we respectfully disagree with the reviewer that it is known that these vertex-specific molecules mark a material point with tricellular adhesion. This is something that is often assumed but has not been tested. In fact, Sidekick adhesion molecules form trans dimers and, in flies, we and others have demonstrated that its presence in only two cells (not three) is sufficient for its localisation at tricellular vertices (Finegan et al., PLOS Bio (2019) [doi: 10.1371/journal.pbio.3000522](https://doi.org/10.1371/journal.pbio.3000522); Letizia et al., Dev Cell (2019) doi: 10.1016/j.devcel.2019.07.007 ). So it it unlikely that Sidekick forms trimers, for example, that could conceivably “pin” the vertices. It is also unknown how Sidekick is localised at vertices in the first place. We are indeed excited about exploring these molecules, but we do not think that there is, as of now, sufficient evidence to impose a specialised vertex adhesion as a material point that pins vertices. This aligns with recent experimental evidence that vertices are not pinned and can “slide” (Vanderleest et al., eLife (2018) doi: [10.7554/eLife.34586](https://doi.org/10.7554/eLife.34586)). We additionally highlight that mutants in sdk (for example) are viable, while E-cadherin mutants are not, justifying a choice of a bicellular adhesion structure in a minimal model. We therefore focus on establishing the model with essential bicellular adhesions, such as E-Cadherins, which paves the way for understanding additional adhesion systems, which might modulate cell intercalation behaviour.

In order to understand the exact mechanical roles of tricellular junction structures, the most likely route is to understand the differences between the wildtype and mutant phenotype. This will require additional theoretical and experimental work beyond the scope of this paper. In addition, as we stress in our introduction, the mechanics of epithelia are so far not explained from the basis of adhesion dynamics, which indeed requires a model like the ACAM to be understood.

In addition, the fact that active contractility in vertical junctions causes contractions of the junction seems really trivial; or are authors simply stating here that their more fine-grained model reproduces the behavior of the biological model (as well as the existing simpler single-edge model)? The same applies to section 2.10 (bicellular junctions have asymmetric contractility); is this simply a reproduction of the feature already known from the single-edge model in Rauzi et al. 2008, i.e. when you introduce a feature into the model that prevents or resists re-elongation of the contracted vertical junction, this promotes successful elongation of horizontal junctions?

Indeed, shrinkage from active contractility is an expected result (as with fracture). However, by lowering the level of phenomenology (compared to e.g. vertex models, including the one in Rauzi et al. 2008), the ACAM has the potential to explain the cell behaviours in terms of the measurable properties of subcellular constituents: actomyosin and adhesion turnover times, crucially. In vertex models, behaviours can be described only in terms of higher-level phenomenological properties (elasticity of cell-level area and perimeter). This also allows us to more directly explain emergent phenotypes in terms of mechanical properties at the subcellular scale. In this sense, the novelty is not so much at the macro (cellular) scale but rather at the micro (subcellular) scale in the model.

The ability to handle asymmetric contractility is one of the novelties that are possible for an apposed cortex model. We believe that our model is unique in being able to approach such a case (where mechanical properties differ across bicellular junctions, Fig 5C). Shared-edge vertex models cannot directly implement this situation, requiring phenomenological additions to the energy of contractile junctions (Tetley et al., eLife (2016) doi: [10.7554/eLife.12094](https://doi.org/10.7554/eLife.12094)), and we provide new theory for solving this problem. Again, the phenomenology obtained at cell scale is not the novelty, but the input to obtain it crucially differs with the one of existing (e.g. vertex) models.

In section 2.6, the authors describe passive extension (“due to decreasing free energy up to an internal angle of 2*pi/3”); is this actually the case experimentally, that the extension proceeds up to an internal angle 2*pi/3? And, again, this passive elongation has already been shown to work in single-edge models, it is not surprising that it would work similarly in this model; but more importantly, Collinet et al. 2015 have already shown compelling evidence that there are likely active mechanisms for horizontal junction elongation; therefore, how does the passive elongation in the apposed-cortex model contribute to our understanding of the biological system?

In the highly active germ-band, the cells do not return to a 2pi/3 configuration because of how active the tissue is; cells pull on each other during intercalation and there is also an antero-posterior tissue-level pull.

We are not claiming that there is novelty in the fact that passive extension is a possibility, or that it does lead to an equilibrium arrangement with 2pi/3 angles. What is novel is to actually simulate the transition from a 4-way configuration to this passive extension. In vertex models, the 4-way configuration has to be “jumped over” because there is a topological change associated with the neighbour exchange. In our model, it is actually simulated. We uniquely show how maintaining the cells to be connected sufficiently close to one another is instrumental in allowing adhesions to appear between them, and then allowing the known passive adhesion mechanism to occur.

In addition, while newly built edges can extend in vertex models, we wish to highlight that vertex models often show repeated “flipping” behaviour when the T1 is actively driven and the surrounding tissue is not pulling on the new edge (as is the case in our simulation). This is sometimes solved by initialising the new edges to be relatively long, but it is not a fair representation of extension to have pre-built the edge with significant length. We show that passive extension happens from the moment that the first adhesion bond forms between the cells coming into contact. As above, we describe the cell behaviour in terms of the micro/subcellular-scale.

An additional novelty of our model is that we give a rate related to the adhesion timescale for this passive extension, which has not previously been shown.

We are certainly excited to apply our model to specific systems and test the contributions of the active mechanisms highlighted by several publications on Drosophila germ-band extension, such as medial contractions and tissue-level forces. However, for this paper, we focused on identifying the minimal ingredients, at the subcellular scale, of an actively driven T1, from which we provide the result that the resolution of the 4-way configuration leading to passive extension can in fact be done with no extra ingredient added to the simple dynamics of dimeric cell-cell adhesions (and does not result in flipping).

In section 2.7 (Adhesion turnover modulates cell area loss), the authors seem to be ignoring a body of literature (starting with Fernandez-Gonzalez 2011) that has shown that cell areas during germ band elongation undergo systematic oscillations. Isn’t it possible that the observed area loss here is merely an alignment artifact? This would mean that the experimentally observed area reduction is not necessarily a specific property of the four-way vertex resolution itself, but part of a continuum of dynamic area changes that occurs throughout the entire vertical junction contraction and horizontal junction elongation.

We thank the reviewer for pointing out the importance of discussing pulsatility in the context of our paper.

Indeed, it is known that intercalating cells in epithelia can exhibit area fluctuations, which have been shown to be associated with medial Myosin contractility (from the Fernandez-Gonzalez 2011 paper and subsequent ones), whereas on average cell area in the germband does not show a systematic trend (Collinet 2015, Fig 2F). However, when averaging cell area specifically when cells are losing a junction during convergent–extension in the Drosophila germ-band, as a function of the length of the shrinking junction, we find that there is also a trend of area reduction to which the model prediction is strikingly similar in magnitude (Figure 4A). Note that the time period typically associated with such a process is typically 10 to 20 minutes. We find indeed that both in our germ-band measurements and in the model, this seems to be a continuous process throughout vertical junction contraction and can be explained in terms of the properties of adhesions in the model.

The role of medial Myosin in the tissues where pulsations are observed is of course of high interest, and we have added a discussion in both section 2.7 and the introduction. However, for this paper, we have chosen not to include them in the model since we would like to highlight the potential for junctional-only mechanisms to produce relevant T1 dynamics and possible implication for area variations.

# Reviewer #3:

Summary: In this manuscript, the authors present a new computational model for describing the behaviors of intercalating epithelial cells during processes such as convergent extension. Their model, which they call the “apposed cortex adhesion model” (ACAM), represents a considerable advance over older junction-vertex models. In older models, complex cell-cell interfaces were represented as single springs connecting nodes; and while these models could often recapitulate the phenomenological behaviors of intercalating cells, they were not very informative about the underlying causes of these behaviors. With ACAM, the authors created a two-dimensional model in which each actomyosin cortex is represented as a continuous rope-like structure with differential adhesion between neighboring cells. As their model parameters have actual subcellular processes (e.g., myosin contraction and membrane turnover) as analogs, this allows them to use this model to better predict how cells will behave in different contexts, and which subcellular processes are important for which behaviors. While the manuscript was written with Drosophila germband extension in mind, this model should be applicable to other cellular contexts as well. Importantly, the authors do make several interesting predictions/conclusions using this model. 1) They show that intercalation can be explained completely by junctional behaviors, and that new edge formation does not require an active process. 2) They are able to explain decreases in apical cell area during intercalation in terms of increased slippage between apposed cortices, which to me is a novel prediction. And 3) They are able to recapitulate rosette formation under certain conditions with high adhesion friction, suggesting that rosettes are really a result of active edge contraction winning out over passive new edge formation.

We are very appreciative of this summary of our work, which we feel accurately captures both our intentions and results. Thank you.

Issues:

My major issue with the paper (if it can be called a major issue) is the question of whether medial myosin forces need to be incorporated into the model. I understand this would be a major change to the model, and it is probably beyond the scope of a revision. Upon reading the Discussion section again, I do believe that they address the most obvious questions concerning how medial myosin might affect the model.

We certainly share the reviewer’s interest in questions surrounding medial Myosin. However, as for other features only described so far for germband extension (such as stress-dependent behaviour of E-Cadherins or anterio-posterior tissue-scale pulling force), we have made the choice not to include these features in this first version of the ACAM. Because we introduce a model that is quite different to existing ones, we were interested in deriving the minimal conditions required for active neighbour exchanges, using ingredients that are known to be common across tissue types. We found that medial forces were not a necessary requirement of the process and therefore did not include this tissue-specific feature in our first presentation of the model.

The model is, however, in a good position to incorporate medial Myosin in a less phenomenological way than for example vertex models. This is a high priority for future work, to be tied with additional experimental data from the germ-band.

Line 43. The authors use the phrase “can cortical actomyosin flow past a cell vertex into neighboring junctions…” (or some similar phrase), several times throughout the paper. I’m not sure I understand what this phrase means. Are they asking whether the vertex is a physical boundary that stops myosin from moving past it? Or are they asking whether myosin can move along paths other than the junctions? They should rephrase here and throughout the paper to make their meaning more clear.

Yes, indeed, we are asking whether the vertex is a physical boundary that stops actomyosin or adhesion molecules to move past it. We think this is an interesting question highlighted by our model. The point that we want to make is indeed whether there is an obstacle at the subcellular level for actomyosin to “change neighbour”, or not, which can be seen as either the vertex “sliding along” the cell junctional cortex, or, changing viewpoint, as the actomyosin moving within the cell past the vertex. Since our model for actomyosin is liquid-like at the timescale of rearrangements, we have named this “flow past a vertex”. Note in addition that, in this paper, we consider only actual flow of the (liquid-like) actomyosin cortex, which is not blocked by a physical boundary at the vertex but can, as we show, be prevented from “flowing freely” past it because of slow adhesion turnover. Other routes of transport, via e.g. trafficked vesicles may contribute and are interesting outlets to explore in future work.

We have found it difficult to describe concisely and unambiguously this motion, which we believe is best visualized in movie 3B. We have amended the sentence in the introduction and rephrased elsewhere in the hope to make this entirely clear.

Line 50: I think the authors are being a bit hyperbolic when they state “However, the physical roles that these subcellular features take and whether they are strictly necessary or merely supportive, are not known.” First, the word “features” is too vague. If they are referring to myosin, as there has been an immense amount of work showing that myosin is necessary and sufficient for cell intercalation in many systems, so this statement isn’t true. I think it would help the paper if they reworded this paragraph to be more specific about what is truly unknown at this point.

We have reworded this section to be more specific.

Minor issues:
Line 78, 86, etc: Should “continuum” be “continuous”? It sounds a bit strange…

Indeed, we have used “continuous” instead in those places.

Figure 4E. Is the top axis label correct? Should it read: {1,9} {1,7} {1,3} {1,2} {1,4} {1,11} {1,9}

Yes, this is an error. Thank you.
